# Clinical Characteristics and Influencing Factors of Depressive Symptoms in Patients With Vascular Cognitive Impairment

**DOI:** 10.62641/aep.v54i3.2194

**Published:** 2026-06-15

**Authors:** Hui Sun, Jingjing Ye, Yuyu Li, Li Xu

**Affiliations:** ^1^Department of Neurology, The First Affiliated Hospital of Bengbu Medical University, 233004 Bengbu, Anhui, China; ^2^Clinical Trial Research Center, The First Affiliated Hospital of Bengbu Medical University, 233004 Bengbu, Anhui, China

**Keywords:** vascular cognitive impairment, depressive symptoms, lesion location, anatomical specificity, age-related patterns, treatment response prediction

## Abstract

**Background:**

This study aimed to characterize depressive symptoms in patients with vascular cognitive impairment (VCI) after ischemic stroke and to identify independent predictors of treatment response to antidepressant therapy, with a focus on lesion-location heterogeneity.

**Methods:**

This retrospective observational cohort study enrolled 224 patients with VCI and concomitant depressive symptoms from June 2022 to June 2024. Depression severity was assessed using the 17-item Hamilton Depression Rating Scale (HAMD-17). All patients completed 8 weeks of standardized treatment including antidepressant medication and cognitive rehabilitation. Treatment response was defined as ≥50% reduction in HAMD-17 from baseline to week 8. Lesion locations were categorized into eight mutually exclusive anatomical groups (frontal, temporal, parietal, occipital, basal ganglia/internal capsule, thalamus, pons and cerebellum). Univariate and multivariable logistic regression identified predictors of treatment response, and Receiver Operating Characteristic (ROC) analysis evaluated model performance.

**Results:**

Mean baseline HAMD-17 was 27.4 ± 3.1, and 65.2% (146/224) achieved treatment response. Baseline depressive symptom severity differed significantly across lesion locations (one-way analysis of variance (ANOVA): F (7,216) = 3.48, *p* = 0.001), whereas baseline anxiety severity did not (F (7,216) = 0.72, *p* = 0.652). In multivariable analysis, lower baseline Hamilton Anxiety Rating Scale (HAMA) score (odds ratio (OR) = 0.93, 95% confidence interval (CI): 0.88–0.98, *p* = 0.006), shorter time since stroke (OR = 0.86 per month, 95% CI: 0.75 to 0.99, *p* = 0.034), and higher education-adjusted Montreal Cognitive Assessment (MoCA) score (OR = 1.12 per point, 95% CI: 1.01–1.24, *p* = 0.031) were independently associated with treatment response. The prediction model demonstrated moderate discriminative ability (area under the curve (AUC) = 0.795, 95% CI: 0.738–0.851), with sensitivity of 0.890 and specificity of 0.608 at the optimal cutoff.

**Conclusions:**

Depressive symptom burden in post-stroke VCI exhibits significant anatomical heterogeneity across lesion locations. Baseline anxiety severity, disease duration, and baseline cognitive performance moderately predict treatment response, supporting early risk stratification and individualized management.

## Introduction

Vascular cognitive impairment (VCI) is a clinical syndrome of varying cognitive decline due to cerebrovascular pathology and is the second most common cause of dementia after Alzheimer’s disease [1, 2]. The Global Burden of Disease study shows rising global incidence of cerebrovascular disease, severely impacting patient quality of life and clinical prognosis [3, 4]. In China, with accelerating population aging, VCI prevalence has increased annually, imposing substantial economic burdens on families and healthcare systems [5, 6]. The heterogeneous nature of VCI, encompassing various pathological substrates including large vessel disease, small vessel disease, and strategic infarcts, contributes to the complexity of its clinical presentation and therapeutic management [7].

Depressive symptoms are among the most frequent neuropsychiatric complications of VCI. Epidemiological studies suggest that approximately 35–50% of patients with VCI experience comorbid depression, a rate markedly higher than in the general population [8, 9]. The co-occurrence of VCI and depression worsens clinical outcomes by aggravating cognitive decline, impairing functional recovery, reducing treatment adherence and increasing long-term mortality [10, 11, 12]. Consequently, identifying factors associated with treatment response in VCI patients with depressive symptoms has critical clinical relevance [13].

The pathophysiology of depression in VCI is multifactorial, involving disrupted mood-regulating neural circuits, neuroinflammatory activation, and neurotransmitter dysregulation [14, 15, 16, 17]. Strategic infarcts affecting cortico-striato-thalamo-cortical pathways may predispose patients to depressive symptoms, while inflammatory mediators such as interleukin-6 (IL-6) and tumour necrosis factor-α further impair neurotransmission and neuroplasticity [18]. These mechanisms may also contribute to the variable and often suboptimal response to standard antidepressant therapy observed in this population.

Current management of VCI with depression relies primarily on selective serotonin reuptake inhibitors and supportive rehabilitation [19]. However, delayed efficacy onset, adverse effects and treatment resistance remain common, with approximately one-third of patients failing to achieve an adequate response [20]. Current management includes antidepressant treatment, vascular risk-factor control and rehabilitation-based supportive interventions. However, treatment response remains heterogeneous, and robust evidence on predictors of short-term response in real-world patients with post-stroke VCI is still limited. Therefore, identifying clinically accessible baseline factors associated with treatment response may help improve early risk stratification and individualized management [21].

We conducted a retrospective cohort study of 224 patients with post-stroke VCI and depressive symptoms to evaluate treatment response after 8 weeks of standardized therapy. The primary aim was to identify independent predictors of treatment response using multivariable logistic regression and to establish a clinically applicable prediction model with Receiver Operating Characteristic (ROC)-based performance assessment. In addition, we examined whether depressive symptom severity differed across infarct lesion locations, providing anatomical evidence relevant to vascular depression in VCI. These findings may help improve early risk stratification and support individualized management for this complex patient population.

## Materials and Methods

### Study Design and Data Source

This retrospective observational cohort study investigated clinical characteristics, anatomical patterns and predictors of depressive symptoms in patients with VCI. Clinical data were collected from patients hospitalized or receiving outpatient treatment in the Department of Neurology at The First Affiliated Hospital of Bengbu Medical University from June 2022 to June 2024. Data were extracted from the hospital’s electronic medical record system, including demographic information, diagnostic details, imaging findings, neurological assessments, psychiatric evaluations, treatment records and laboratory results. This study was approved by the Ethics Committee of The First Affiliated Hospital of Bengbu Medical University (Approval No. [2025] 049) and complied with the Declaration of Helsinki. Written informed consent was obtained from all patients or their legal guardians.

### Inclusion and Exclusion Criteria

The inclusion criteria were set as follows: (1) diagnosis of VCI according to VasCog-2-WSO criteria, supported by Computed Tomography (CT) or Magnetic Resonance Imaging (MRI) evidence of cerebrovascular lesions [22]; (2) depressive disorder diagnosed according to Diagnostic and Statistical Manual of Mental Disorders, Fifth Edition (DSM-5) criteria after the index ischemic stroke [23]; (3) baseline 17-item Hamilton Depression Rating Scale (HAMD-17) score ≥24, indicating severe depressive symptom [24] (not as a diagnostic criterion) [25]; (4) ischemic stroke 3–9 months prior to enrolment, with persistent cognitive complaints or objective impairment at assessment; (5) cognitive impairment for eligibility screening was operationally defined as a baseline Montreal Cognitive Assessment (MoCA) score of 18–25. This cutoff was used as a study-specific screening threshold to include patients with non-dementia-level VCI and was not intended as a formal severity grading criterion [26]; (6) age 40–75 years; and (7) Completion of 8 weeks of standardized treatment with available baseline and follow-up psychiatric assessment data.

The exclusion criteria were as follows: (1) history of intracerebral haemorrhage, transient ischemic attack, or non-ischemic cerebrovascular disorders; (2) cognitive decline clearly preceding the index stroke or clinical/imaging features suggestive of primary neurodegenerative disorders (e.g., Alzheimer’s disease, Lewy body dementia, or frontotemporal dementia); (3) pre-existing major psychiatric disorders that clearly predated the index stroke (including major depressive disorder, bipolar disorder, schizophrenia, or anxiety disorders) or long-term psychotropic medication use unrelated to post-stroke symptoms; (4) severe systemic diseases (e.g., advanced cardiac, hepatic, renal dysfunction, or active malignancy) that could significantly affect prognosis or psychiatric assessment; (5) patients with more severe cognitive deficits, operationally defined in this study as MoCA <18, were excluded because such impairment was considered likely to compromise the reliability and completeness of psychiatric assessment. This threshold was used for study feasibility and assessment reliability rather than as a universally accepted diagnostic cutoff for severe cognitive impairment [26, 27]; (6) treatment interruption exceeding 1 week during the observation period; or (7) incomplete medical records or missing key baseline or follow-up data.

### Clinical Assessment and Data Collection

#### Demographic and Clinical Variables

Baseline demographic characteristics were obtained from the electronic medical record system, including sex, age, body mass index, years of education, smoking status and alcohol consumption). Clinical data included time elapsed since ischemic stroke (months), prior cerebrovascular events and vascular comorbidities (e.g., hypertension and diabetes). All comorbid conditions were diagnosed according to standard clinical criteria and documented in the medical records. VCI was diagnosed in accordance with contemporary consensus recommendations for vascular-related cognitive disorders. The diagnosis required measurable cognitive decline together with neuroimaging evidence of cerebrovascular pathology and clinical judgment supporting a vascular contribution to cognitive dysfunction.

Cognitive impairment was initially screened using the MoCA. Routine neurological evaluations in our department included structured assessment of executive function, attention, memory performance, language abilities and visuospatial skills. Patients were categorized as having mild-to-moderate VCI when cognitive deficits were evident but daily functional independence was largely preserved and diagnostic criteria for dementia were not fulfilled [28].

To minimize diagnostic overlap with primary neurodegenerative conditions, we excluded patients whose cognitive decline clearly preceded the index stroke or whose imaging demonstrated patterns more consistent with degenerative disorders (e.g., disproportionate medial temporal atrophy relative to vascular lesion burden). Clinical features suggestive of Lewy body dementia, frontotemporal dementia, or other non-vascular neurodegenerative syndromes led to exclusion [29]. Uncertain cases were reviewed jointly by senior neurologists to ensure classification consistency.

#### Neurological Assessment

Baseline stroke severity was assessed using the National Institutes of Health Stroke Scale (NIHSS) [30], a 15-item neurological deficit scale with a total score ranging from 0 to 42, with higher scores indicating greater neurological impairment. Assessed domains include level of consciousness, level of consciousness questions, level of consciousness, best gaze, visual fields, facial palsy, motor arm, motor leg, limb ataxia, sensory function, best language, dysarthria and extinction/inattention. For descriptive categorization, NIHSS scores were classified as 1–4 (minor stroke), 5–15 (moderate), 16–20 (moderate-to-severe) and 21–42 (severe) [31]. The NIHSS has acceptable reliability (e.g., the Persian validation Cronbach’s α coefficient of 0.81) [32].

Lesion location was determined by neuroimaging (e.g., CT or MRI) and classified into eight anatomical categories: (1) frontal lobe, (2) temporal lobe, (3) parietal lobe, (4) occipital lobe, (5) basal ganglia/internal capsule, (6) thalamus, (7) pons, and (8) cerebellum. For patients with multiple infarcts in different vascular territories, classification was based on the clinically predominant lesion, defined as the largest lesion volume and/or the lesion most closely associated with presenting neurological deficits. Cases with diffuse small vessel changes without a clearly dominant lesion were excluded from lesion-specific analyses [33].

Lesion volume was calculated using standard radiological methods and expressed in millilitres (mL). All neuroimaging interpretations were performed by experienced neuroradiologists blinded to patient psychiatric status. Cognitive function was assessed with MoCA (0–30) at baseline and at week 8 [26]. For individuals with ≤12 years of education, one point was added to the total MoCA score in accordance with recommended correction procedures. MoCA was used for eligibility screening and outcome evaluation.

#### Psychiatric Assessment

Depression severity was evaluated using HAMD-17 (0–52) [25, 34], and anxiety severity with Hamilton Anxiety Rating Scale (HAMA, 0–56) [35]. Assessments were performed at baseline and week 8 by trained neurologists/psychiatrists blinded to imaging classification when feasible. Clinically significant anxiety was defined as HAMA ≥ 14 [36]. Anxiety–depression comorbidity was defined as the presence of depressive disorder diagnosed according to DSM-5 criteria together with clinically significant anxiety (HAMA ≥ 14) at baseline.

#### Biomarker Measurements

Fasting venous blood samples were collected at baseline and week 8. Serum levels of brain-derived neurotrophic factor (BDNF), 5-hydroxytryptamine (5-HT), norepinephrine (NE), IL-6, tumor necrosis factor-α (TNF-α), and high-sensitivity C-reactive protein (hs-CRP) were measured using enzyme-linked immunosorbent assay (ELISA) kits (BDNF, 5-HT, NE: Wuhan Elabscience Biotechnology Co., Ltd., Wuhan, China; IL-6, TNF-α, hs-CRP: Jiangsu Meimian Industrial Co., Ltd., Yancheng, China) according to manufacturer instructions.

BDNF, 5-HT and NE concentrations were expressed in ng/mL; IL-6 and TNF-α in pg/mL; and hs-CRP in mg/L. The lower limits of detection were 0.1 ng/mL for BDNF and monoamines, 0.5 pg/mL for IL-6 and TNF-α and 0.1 mg/L for hs-CRP [37]. Intra- and inter-assay coefficients of variation were <10%. All assays were performed in duplicate, and laboratory personnel were blinded to treatment response classification. Baseline and week 8 samples from the same patient were analysed within the same batch to minimize inter-assay variability.

### Treatment Protocol

All patients received standardized 8-week treatment including: (1) guideline-based vascular management; (2) antidepressant pharmacotherapy (escitalopram 10–20 mg once daily with titration based on tolerability) [38]; and (3) cognitive rehabilitation sessions. Treatment adherence was verified by chart review. Analyses were performed using IBM SPSS Statistics for Windows (version 26.0; IBM Corp., Armonk, NY, USA) and R software (version 4.2.0; R Foundation for Statistical Computing, Vienna, Austria).

### Outcome Measures

Primary outcome was treatment response, defined as ≥50% reduction in HAMD-17 score from baseline to week 8. Secondary outcomes changes in HAMD-17, HAMA, education-adjusted MoCA, and biomarker levels [39]. For continuous outcomes, change scores were calculated as week 8 minus baseline values. Patients were classified as responders or non-responders accordingly [40].

### Statistical Analysis

Analyses were performed using IBM SPSS Statistics for Windows and R software. Normality of continuous variables was assessed using the Shapiro–Wilk test. Normally distributed data were presented as mean ± standard deviation, whereas non-normally distributed variables were expressed as median (interquartile range (IQR)). For within-subject before–after comparisons (baseline vs week 8), paired sample t-tests or Wilcoxon signed-rank tests were applied as appropriate. Between-group comparisons (responders vs. non-responders) were conducted using independent sample t-tests or Mann-Whitney U tests for continuous variables and χ^2^ or Fisher’s exact tests for categorical variables. Baseline characteristics were first compared between responders and non-responders, and variables with *p* < 0.10 in univariate analyses were subsequently included in the multivariable logistic regression model. Years of education was not included as a separate variable in the multivariable model because MoCA score had already been adjusted for education, which partially accounts for the effect of educational level. For comparisons across multiple lesion-location groups, one-way analysis of variance (ANOVA) or Kruskal-Wallis tests were used as appropriate. Univariate logistic regression identified candidate predictors of treatment response, and variables with *p* < 0.10 were entered into a multivariable logistic regression model using the enter method. Results were reported as odds ratios with 95% confidence intervals (CIs). Serum biomarkers and lesion location were not included in the multivariable model because they were not significantly associated with treatment response in univariate analyses and did not meet the predefined inclusion criterion (*p* < 0.10). In addition, lesion location is a multi-category variable, which may introduce model instability given the sample size. Model discrimination and calibration were assessed using ROC curve analysis and the Hosmer-Lemeshow test. Multicollinearity was evaluated using variance inflation factors (VIF), with all VIF values < 2.0. Only patients with complete baseline and follow-up data were included in the final analysis.

## Results

### General Characteristics of Study Subjects

A total of 224 patients with ischemic stroke VCI (cerebral infarction) were included. All were evaluated ≥3 months post-stroke with persistent cognitive complaints ≥3 months. All met operational criteria for post-stroke VCI (VCI spectrum) not post-stroke dementia. Cognitive function was primarily described using MoCA (0–30); education-adjusted MoCA added 1 point for ≤12 years of education. Of 224 participants, 136 (60.7%) were male and 88 (39.3%) were female. The mean age was 61.4 ± 7.6 years. Time since stroke ranged from 3 to 9 months, with a mean of 4.9 ± 1.4 months. Hypertension and diabetes were present in 141 (62.9%) and 82 (36.6%) patients, respectively. Baseline scores were: MoCA raw 19.6 ± 1.6, MoCA education-adjusted 20.3 ± 1.8, HAMA 22.2 ± 5.3, and HAMD-17 27.4 ± 3.1 (indicating severe depressive symptoms). Baseline serum biomarker levels were also measured, including BDNF, 5-HT, NE, IL-6, TNF-α and hs-CRP (Table 1).

**Table 1.** **Baseline characteristics of the study population (n = 224)**.

Variable	Total (n = 224)
Sex (male/female), n	136/88
Age, years (mean ± SD)	61.4 ± 7.6
Time since stroke, months (mean ± SD)	4.9 ± 1.4
Hypertension, n (%)	141 (62.9)
Diabetes, n (%)	82 (36.6)
MoCA raw (0–30), mean ± SD	19.6 ± 1.6
MoCA education-adjusted† (0–30), mean ± SD	20.3 ± 1.8
HAMA (0–56), mean ± SD	22.2 ± 5.3
HAMD-17 (0–52), mean ± SD	27.4± 3.1
Years of education, years (mean ± SD)	9.8 ± 3.4
BDNF (ng/mL), mean ± SD	18.3 ± 4.1
5-HT (ng/mL), mean ± SD	91.3 ± 18.2
NE (ng/mL), mean ± SD	0.47 ± 0.09
IL-6 (pg/mL), mean ± SD	6.7 ± 2.4
TNF-α (pg/mL), mean ± SD	8.0 ± 2.6
hs-CRP (mg/L), mean ± SD	4.1 ± 1.5

Note: MoCA, Montreal Cognitive Assessment; HAMA, Hamilton Anxiety Rating Scale; HAMD-17, the 17-item Hamilton Depression Rating Scale; BDNF, brain-derived neurotrophic factor; 5-HT, 5-hydroxytryptamine; NE, norepinephrine; IL-6, interleukin-6; TNF-α, tumor necrosis factor-α; hs-CRP, high-sensitivity C-reactive protein. †MoCA education correction: +1 point for individuals with ≤12 years of education.

### Comparison of Treatment Efficacy

**Fig. 1.**
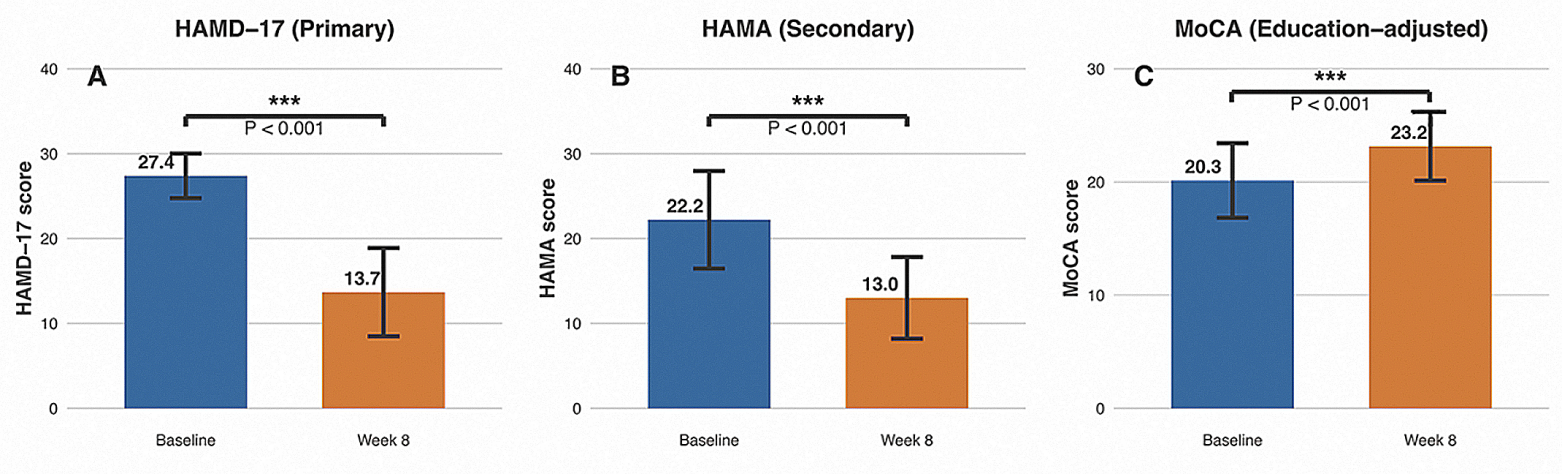
**Changes in depressive symptoms, anxiety symptoms and cognitive function from baseline to week 8**. Treatment response was defined as ≥50% reduction in HAMD-17 from baseline to week 8; 146/224 patients (65.2%) were classified as responders and 78/224 (34.8%) as non-responders. (A) Depressive symptoms (primary outcome): HAMD-17 decreased from 27.4 ± 3.1 at baseline to 13.7 ± 5.2 at week 8 (mean change -13.4 ± 5.1, *p* < 0.001). (B) Anxiety symptoms (secondary outcome): HAMA decreased from 22.2 ± 5.3 to 13.0 ± 5.0 (mean change -9.2 ± 4.6, *p* < 0.001). (C) Cognitive performance: education-adjusted MoCA increased from 20.3 ± 1.8 to 23.2 ± 3.0 (mean change 3.1 ± 2.1, *p* < 0.001). Data are presented as mean ± SD. *p* values indicate within-group comparisons between baseline and week 8. HAMD-17, 17-item Hamilton Depression Rating Scale; HAMA, Hamilton Anxiety Rating Scale; MoCA, Montreal Cognitive Assessment; SD, standard deviation; ***p < 0.001.

After 8 weeks of standardized treatment, 146 of 224 patients (65.2%) achieved a treatment response (≥50% reduction in HAMD-17); 78 (34.8%) were non-responders. Depressive symptoms, anxiety symptoms, and cognitive performance all improved significantly during follow-up. Mean HAMD-17 score decreased from 27.4 ± 3.1 at baseline to 13.7 ± 5.2 at week 8 (mean change: -13.4 ± 5.1, 95% confidence interval (CI): -14.1 to -12.7; *p* < 0.001). Mean HAMA score decreased from 22.2 ± 5.3 to 13.0 ± 5.0 (mean change: -9.2 ± 4.6, 95% CI: -9.9 to -8.5; *p* < 0.001). By contrast, education-adjusted MoCA increased from 20.3 ± 1.8 to 23.2 ± 3.0 (mean change: 3.1 ± 2.1, 95% CI: 2.7 to 3.5; *p* < 0.001), indicating concurrent cognitive improvement (Fig. 1).

Patients were divided into responders (n = 146) and non-responders (n = 78) based on HAMD-17 reduction. Baseline characteristics were compared between the two groups. Compared with non-responders, responders had significantly shorter time since stroke, lower baseline HAMA scores, lower baseline HAMD-17 scores, and higher education-adjusted MoCA scores. No significant differences were observed in sex, age, or diabetes between the two groups. Hypertension was more frequent in non-responders but not statistically significant (Table 2).

**Table 2.** **Baseline characteristics of responders and non-responders**.

Variable	Responders (n = 146)	Non-responders (n = 78)	*p* value
Male, n (%)	90 (61.6)	46 (59.0)	0.704
Age (years)	60.9 ± 7.4	62.3 ± 7.9	0.089
Time since stroke (months)	4.7 ± 1.3	5.3 ± 1.5	0.008
Hypertension, n (%)	86 (58.9)	55 (70.5)	0.082
Diabetes, n (%)	53 (36.3)	29 (37.2)	0.893
MoCA (education-adjusted)	20.6 ± 1.7	19.8 ± 1.9	0.002
Baseline HAMA score	21.0 ± 4.9	24.4 ± 5.5	<0.001
Baseline HAMD-17 score	27.1 ± 3.0	28.0 ± 3.2	0.036

Note: Data are presented as mean ± SD or n (%). Continuous variables were compared using the independent samples t-test, and categorical variables were compared using the χ^2^ test. MoCA, Montreal Cognitive Assessment; HAMA, Hamilton Anxiety Rating Scale; HAMD-17, the 17-item Hamilton Depression Rating Scale.

### Univariate Analysis of Treatment Response

Univariate logistic regression identified factors associated with depression treatment response. Baseline symptom severity showed negative associations with treatment response: baseline HAMA (odds ratio (OR) = 0.92, 95% CI: 0.88 to 0.96, *p* < 0.001) and baseline HAMD-17 (OR = 0.91, 95% CI: 0.83 to 0.99, *p *= 0.028) indicated that patients with more severe baseline anxiety or depressive symptoms were less likely to achieve treatment response. Longer time since stroke was also negatively associated with response (OR = 0.84 per month, 95% CI: 0.74 to 0.96, *p* < 0.010). Higher baseline cognitive performance (education-adjusted MoCA) was positively associated with response (OR = 1.14 per point, 95% CI: 1.04 to 1.25, *p *= 0.006). In addition, hypertension was significantly associated with a lower likelihood of treatment response (OR = 0.59, 95% CI: 0.35 to 0.99, *p *= 0.046), whereas diabetes was not (*p *= 0.640). Age showed a borderline association (*p *= 0.071). Baseline serum biomarkers (e.g., BDNF, 5-HT, NE, IL-6, TNF-α, and hs-CRP) were not significantly associated with treatment response (all *p* > 0.05), and were not included in the multivariable model according to the predefined selection criterion. Variables with *p* < 0.10 were entered into the multivariable model (Table 3).

**Table 3.** **Univariate logistic regression analysis of depression treatment response**.

Variable	B	SE	OR (95% CI)	Wald	*p* value
Gender (male = 1)	-0.127	0.286	0.88 (0.50–1.54)	0.2	0.657
Age (years)	-0.031	0.017	0.97 (0.94–1.00)	3.28	0.071
Time since stroke (months)	-0.174	0.067	0.84 (0.74–0.96)	6.74	<0.01
Hypertension (yes = 1)	-0.532	0.266	0.59 (0.35–0.99)	4	0.046
Diabetes (yes = 1)	-0.124	0.271	0.88 (0.52–1.50)	0.21	0.64
MoCA (education-adjusted)	0.131	0.047	1.14 (1.04–1.25)	7.78	0.006
Baseline HAMA score	-0.087	0.022	0.92 (0.88–0.96)	15.6	<0.001
Baseline HAMD-17 score	-0.094	0.043	0.91 (0.83–0.99)	4.85	0.028
BDNF (baseline)	0.018	0.021	1.02 (0.98–1.06)	0.73	0.393
5-HT (baseline)	0.006	0.007	1.01 (0.99–1.02)	0.74	0.39
NE (baseline)	0.842	0.665	2.32 (0.63–8.52)	1.62	0.206
IL-6 (baseline)	-0.041	0.048	0.96 (0.88–1.05)	0.73	0.392
TNF-α (baseline)	-0.028	0.041	0.97 (0.90–1.05)	0.47	0.493
hs-CRP (baseline)	-0.067	0.062	0.94 (0.83–1.07)	1.17	0.279

Note: Variables with *p* < 0.10 in univariate logistic regression were included in the multivariable analysis. Baseline serum biomarkers were analysed but not included in the multivariable model due to lack of statistical significance. MoCA, Montreal Cognitive Assessment; HAMA, Hamilton Anxiety Rating Scale; HAMD-17, the 17-item Hamilton Depression Rating Scale; BDNF, brain-derived neurotrophic factor; 5-HT, 5-hydroxytryptamine; NE, norepinephrine; IL-6, interleukin-6; TNF-α, tumor necrosis factor-α; hs-CRP, high-sensitivity C-reactive protein; OR, odds ratio; CI, confidence interval; B, beta; SE, standard error.

### Multivariable Logistic Regression Analysis of Treatment Response

Variables with *p* < 0.1 in univariate analysis were included in the multivariable logistic regression model to identify independent predictors of depression treatment response. After adjustment, lower baseline HAMA score (OR = 0.93, 95% CI: 0.88 to 0.98, *p* = 0.006), shorter time since stroke (OR = 0.86 per month, 95% CI: 0.75 to 0.99, *p* = 0.034), and higher education-adjusted MoCA score (OR = 1.12 per point, 95% CI: 1.01 to 1.24, *p = *0.031) remained independent predictors of treatment response. Age and baseline HAMD-17 were retained in the multivariable model according to the prespecified variable-selection rule but did not reach statistical significance after adjustment (Table 4).

**Table 4.** **Multivariable logistic regression analysis of treatment response**.

Variable	B	SE	OR (95%CI)	Wald	*p* value
Age (per 1-year increase)	–0.019	0.019	0.98 (0.94–1.02)	1.02	0.312
Time since stroke (months)	–0.151	0.072	0.86 (0.75–0.99)	4.49	0.034
Hypertension (yes = 1)	–0.446	0.289	0.64 (0.36–1.13)	2.37	0.123
MoCA (education-adjusted)	0.113	0.052	1.12 (1.01–1.24)	4.66	0.031
Baseline HAMA score	–0.072	0.026	0.93 (0.88–0.98)	7.61	0.006
Baseline HAMD-17 score	–0.051	0.049	0.95 (0.86–1.05)	1.09	0.296

Note: Model χ^2^ = 41.8, *p* < 0.001; Hosmer–Lemeshow test χ^2^ = 6.12, *p* = 0.52; Nagelkerke R^2^ = 0.24. MoCA, Montreal Cognitive Assessment; HAMA, Hamilton Anxiety Rating Scale; HAMD-17, the 17-item Hamilton Depression Rating Scale; OR, odds ratio; CI, confidence interval; B, beta; SE, standard error.

### ROC Curve Analysis of the Predictive Model

**Fig. 2.**
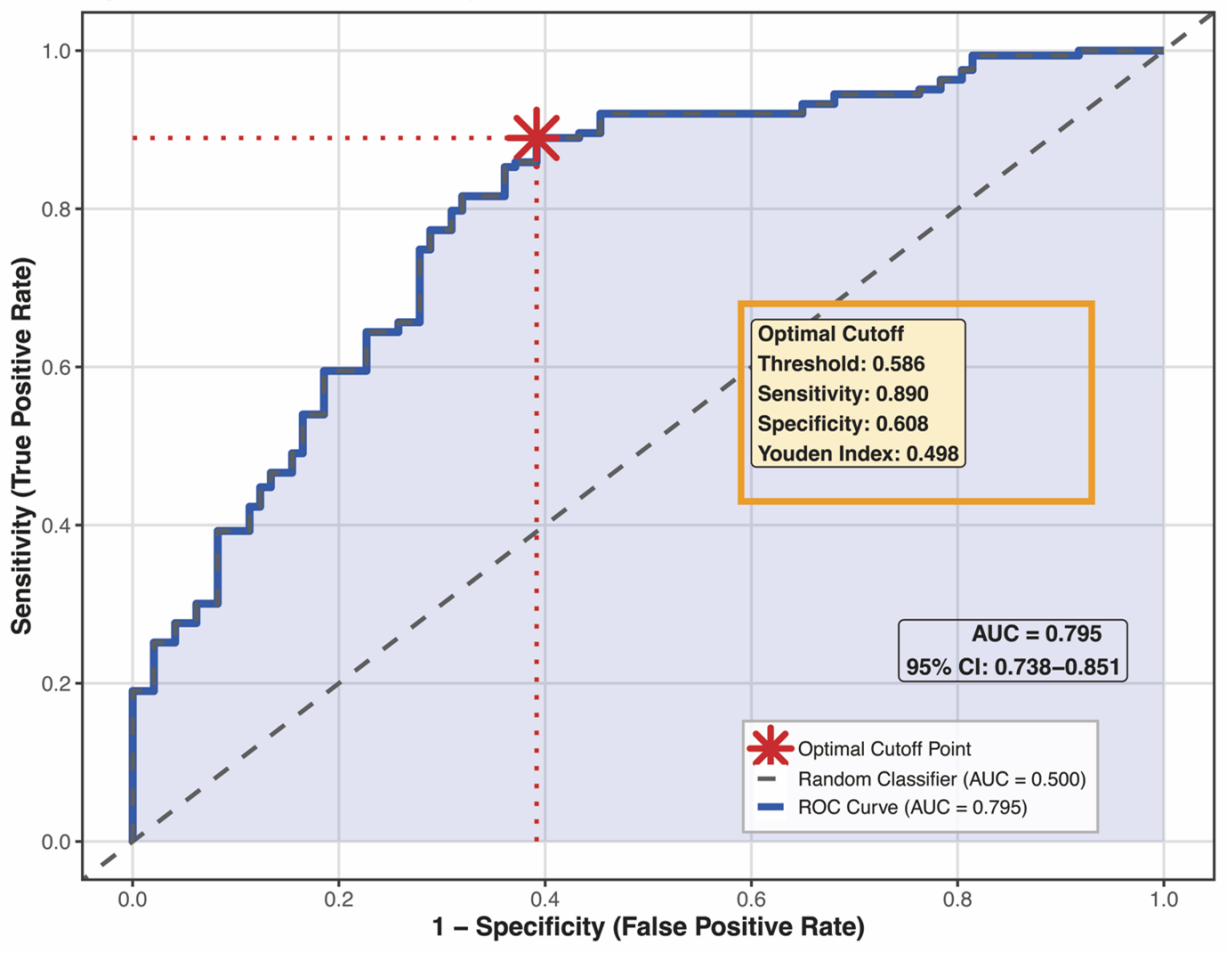
**ROC curve analysis of the treatment response prediction model**. The ROC curve illustrates the discriminative performance of the multivariable logistic regression model for identifying responders (defined as HAMD-17 reduction ≥50% at week 8). The AUC was 0.795 (95% CI: 0.738 to 0.851). The optimal cutoff (maximum Youden index = 0.498) is indicated. HAMD-17, the 17-item Hamilton Depression Rating Scale; ROC, Receiver Operating Characteristic; AUC, area under the curve; CI, confidence interval.

The binary logistic regression analyses were used to determine the predictive A treatment response prediction model was constructed based on the multivariable logistic regression model, and an ROC curve was plotted. The area under the curve was 0.795 (95% CI: 0.738 to 0.851), indicating moderate discriminative ability. At the optimal cutoff (maximum Youden index = 0.498), sensitivity was 0.890 and specificity was 0.608 (Fig. 2).

### Lesion Location and Psychiatric Symptom Profiles

Among the 224 patients, infarct lesions were categorized into eight mutually exclusive anatomical locations (frontal, temporal, parietal, occipital, basal ganglia/internal capsule, thalamus, pons and cerebellum). Baseline depressive symptom severity (HAMD-17) differed significantly across lesion locations (one-way ANOVA: F (7,216) = 3.48, *p* = 0.001), indicating anatomical heterogeneity in depressive symptom burden. Baseline anxiety severity (HAMA) showed no significant variation by lesion location (one-way ANOVA: F (7,216) = 0.72, *p* = 0.652). Stroke severity (NIHSS) also differed significantly among lesion locations (one-way ANOVA: F (7,216) = 3.54, *p* = 0.001). By contrast, neither the anxiety–depression comorbidity rate nor the depression treatment response rate differed significantly across lesion locations (χ^2^ test: *p = *0.910 and *p* = 0.883, respectively, Table 5).

**Table 5.** **Detailed comparison of clinical characteristics and psychiatric symptoms by lesion location**.

Location	n (%)	NIHSS (mean ± SD)	HAMA (mean ± SD)	HAMD-17 (mean ± SD)	Comorbid (%)	Response (%)
Frontal	32 (14.3)	6.1 ± 2.2	21.9 ± 5.4	26.3 ± 3.1	65.6	71.9
Temporal	28 (12.5)	5.7 ± 2.0	20.8 ± 5.0	25.8 ± 2.9	60.7	75
Parietal	25 (11.2)	5.4 ± 2.0	20.5 ± 4.8	25.7 ± 2.8	60	76
Occipital	21 (9.4)	5.3 ± 1.9	20.6 ± 4.9	26.0 ± 3.0	66.7	71.4
Basal ganglia/internal capsule	62 (27.7)	7.2 ± 2.5	22.3 ± 5.3	28.2 ± 3.2	68	74.2
Thalamus	30 (13.4)	6.2 ± 2.2	21.8 ± 5.1	27.8 ± 3.1	66.7	70
Pons	16 (7.1)	7.5 ± 2.6	22.0 ± 5.2	27.1 ± 3.0	62.5	75
Cerebellum	10 (4.5)	6.5 ± 2.4	21.6 ± 5.0	26.8 ± 3.1	60	80
Test statistic/*p*	-	F (7,216) = 3.54/0.001	F (7,216) = 0.72/0.652	F (7,216) = 3.48/0.001	χ² = 2.26/0.910	χ² = 1.85/0.883

Note: Lesion locations were analysed as mutually exclusive groups. Continuous variables (NIHSS, HAMA, HAMD-17) were compared using one-way ANOVA. Categorical variables (comorbidity and response) were compared using χ^2^ tests. NIHSS, National Institutes of Health Stroke Scale; HAMA, Hamilton Anxiety Rating Scale; HAMD-17, the 17-item Hamilton Depression Rating Scale; ANOVA, analysis of variance.

### Association Between Serum Biomarkers and Treatment Response

Baseline serum levels of BDNF, 5-HT, NE, IL-6, TNF-α, and hs-CRP did not differ significantly between responders and non-responders (all *p* > 0.05). After 8 weeks of treatment, responders exhibited significantly greater increases in BDNF, 5-HT, and NE than non-responders (all *p* < 0.05). Both groups showed reductions in IL-6, TNF-α, and hs-CRP with a trend toward larger decreases among responders; however, between-group differences for inflammatory markers did not consistently reach statistical significance (Table 6).

**Table 6.** **Serum biomarkers in responders and non-responders**.

Biomarker	Baseline (R vs NR)	*p*	Δ Change (R vs NR)	*p*
BDNF (ng/mL)	18.6 ± 4.2 vs 17.9 ± 4.0	0.213	6.3 ± 3.4 vs 2.9 ± 3.1	<0.001
5-HT (ng/mL)	92.4 ± 18.5 vs 89.6 ± 17.9	0.328	23.3 ± 14.6 vs 12.8 ± 13.2	0.002
NE (ng/mL)	0.48 ± 0.09 vs 0.46 ± 0.08	0.251	0.14 ± 0.08 vs 0.09 ± 0.07	0.018
IL-6 (pg/mL)	6.8 ± 2.4 vs 6.5 ± 2.3	0.442	-1.9 ± 1.6 vs -0.9 ± 1.7	0.067
TNF-α (pg/mL)	8.1 ± 2.7 vs 7.9 ± 2.6	0.603	-1.9 ± 1.8 vs -1.0 ± 1.9	0.089
hs-CRP (mg/L)	4.2 ± 1.5 vs 4.0 ± 1.4	0.491	-1.4 ± 1.2 vs -0.7 ± 1.3	0.072

Note: Data are presented as mean ± SD. Δ indicates week 8–baseline. *p* values were calculated using independent sample t-tests. R, responders; NR, non-responders; BDNF, brain-derived neurotrophic factor; 5-HT, 5-hydroxytryptamine; NE, norepinephrine; IL-6, interleukin-6; TNF-α, tumor necrosis factor-α; hs-CRP, high-sensitivity C-reactive protein.

## Discussion

This retrospective analysis of 224 patients with VCI and depressive symptoms systematically explored factors influencing treatment response. Results demonstrated that baseline anxiety severity, time since stroke, and baseline cognitive performance were independent predictors of treatment response, providing evidence-based support for individualized clinical treatment protocols. These findings underscore the multifactorial nature of treatment response in this complex patient population and highlight the importance of comprehensive pre-treatment assessment.

Combined traditional Chinese medicine (TCM) treatment was recorded as a routine clinical practice variable but was not used as a grouping factor in this observational analysis; therefore, the present results do not support ’combined TCM treatment’ as an independent predictor of treatment response. Mechanistically, the modified Zhufeng Decoction used in this study exerts its primary effects through calming liver wind, promoting blood circulation, resolving stasis, soothing liver, relieving depression, and nourishing the heart to calm the mind, directly addressing the pathophysiology of VCI with depression [41]. Modern pharmacological research indicates that gastrodigenin possesses antioxidant and neuroprotective properties, tanshinone IIA improves microcirculation, saikosaponin has antidepressant effects, and jujuboside increases brain levels of 5-HT and NE [42, 43, 44, 45]. These multi-target actions provide biological plausibility for symptom improvement in clinical practice; however, the present retrospective dataset does not allow causal attribution of response differences to adjunctive TCM.

Baseline HAMA score was negatively associated with treatment response, indicating that greater anxiety burden predicted a lower likelihood of achieving treatment response. This finding aligns with previous research [46] and may have potential clinical relevance. Several factors may partly explain this association, including greater overall neuropsychiatric burden, lower treatment adherence, and a less favourable response to standard pharmacological treatment. Therefore, patients with more prominent baseline anxiety may warrant closer clinical attention and more individualized management.

Time since stroke (months) negatively correlated with treatment response, indicating that longer disease duration predicts poorer outcomes. Chronic anxiety and depression may induce maladaptive neuroplasticity and more stable pathological neural circuits that are difficult to reverse with short-term treatment [47]. This finding emphasizes the importance of early identification and timely intervention for depressive symptoms in VCI.

Notably, comorbid hypertension showed a negative association with treatment response in univariate analysis, but it did not remain statistically significant after multivariable adjustment, indicating that it should be interpreted as a non-significant trend rather than an independent predictor. Hypertension may still contribute to small vessel disease, blood–brain barrier dysfunction, and inflammation, potentially affecting mood regulation and treatment responsiveness [48]. Thus, vascular risk factor management remains essential. Education level was not separately included in the multivariable model because its effect was partially accounted for by the education-adjusted MoCA score.

The biomarker analysis added mechanistic context to treatment response. Responders showed greater increases in neurotrophic and monoaminergic markers and greater attenuation of inflammatory activity, suggesting that neuroplasticity-related recovery and inflammation control may jointly contribute to symptom improvement [49, 50]. Lesion location was associated with baseline depressive burden, with subcortical involvement (particularly basal ganglia/internal capsule and thalamus) showing higher baseline HAMD-17 scores, whereas short-term response rates did not differ across locations, suggesting that anatomical factors may mainly influence initial severity rather than 8-week response. Baseline biomarker and lesion location did not predict treatment response and was not included in the regression model.

This study has several limitations. The retrospective observational single-centre design may introduce selection bias and limit generalizability. Restricting analyses to patients with complete 8-week follow-up could modestly overestimate response. Treatment was non-randomized, and residual confounding cannot be excluded. Short follow-up precludes long-term outcome assessment. Although the prediction model had high sensitivity, its moderate specificity implies possible false-positive classification and warrants external validation. The cohort was limited to subacute stage (3–9 months) with mild cognitive impairment (MoCA 18–25), which may restrict applicability to other VCI stages. Future multicentre prospective studies are needed to externally validate the prediction model and confirm its generalizability.

## Conclusions

Depressive symptoms in VCI demonstrate anatomical heterogeneity, with subcortical lesions—particularly basal ganglia/internal capsule and thalamus—showing higher baseline depressive severity. Treatment response is independently predicted by baseline anxiety severity, time since stroke, and education-adjusted cognitive status (MoCA). These findings support comprehensive baseline assessment and individualized management for VCI patients with depressive symptoms.

## Availability of Data and Materials

The datasets used and/or analyzed during the current study are available from the corresponding author on reasonable request.
